# Molecular Dynamics Simulation of Graphene Oxide Surface-Modified ADN-Based PBX Double-Shell Structure

**DOI:** 10.3390/molecules31050784

**Published:** 2026-02-26

**Authors:** Shimin Zhang, Jiaqi Wen, Hongxia Zhang, Xiaoying Cheng, Jingyu Wang, Baoyun Ye, Chongwei An

**Affiliations:** School of Environment and Safety Engineering, North University of China, Taiyuan 030051, China; 18735375418@163.com (S.Z.);

**Keywords:** molecular dynamics, ADN, binding energy, mechanical properties

## Abstract

Ammonium dinitramide (ADN), a new-generation green high-energy oxidizer, faces application challenges due to its strong hygroscopicity and poor compatibility with polymer binders. This study proposes a double-shell structure with ADN as the core, graphene oxide (GO) as the intermediate layer, and a binder as the outer shell. Molecular dynamics simulations were performed to investigate composite systems using nitrocellulose (NC), cellulose acetate butyrate (CAB), polystyrene (PS), and their blends NC/CAB and NC/PS as binders. The results demonstrate that GO acts as a “molecular double-sided adhesive”, significantly enhancing the interfacial interaction between ADN and the binders. The NC/PS blend binder exhibits the best overall performance, with the binding energy increased by 1.13 times. Analysis revealed that the NC/PS system establishes the strongest intermolecular interactions among ADN, GO, and the binder via mechanisms like π-π stacking and multiple hydrogen bonds. The glass transition temperature reaches 400.93 K, indicating excellent thermal stability and potential safety/reliability. Mechanical property analysis shows that the NC/PS composite system imparts a better comprehensive balance of stiffness, shear performance, and structural isotropy to the ADN-based polymer-bonded explosive (PBX). This research elucidates the enhancement mechanism of GO and the regulation principles of binders at the molecular scale, providing a theoretical foundation for designing high-performance energetic material.

## 1. Introduction

The currently most promising chlorine-free oxidizer, ammonium dinitramide (ADN), was first synthesized in 1971 in Russia (Oleg Lukyanov, Zelinsky Institute of Organic Chemistry). ADN, as a high-energy and environmentally friendly oxidizer [1,2], has emerged as a highly promising substitute for ammonium perchlorate (AP) in next-generation solid propellants and explosives, owing to its significant advantages such as high oxygen balance, high specific impulse, and halogen-free combustion products [3,4,5]. However, the inherent strong hygroscopicity of ADN and its poor compatibility with many polymeric binders severely limit its practical application [6,7,8,9]. These drawbacks can easily lead to phase separation, deterioration of mechanical properties, and interfacial dewetting during the processing and storage of composite materials, directly affecting the structural integrity and service reliability of the grain [10,11,12]. Therefore, constructing a stable, dense coating layer to fundamentally block the contact between ADN and environmental moisture [13,14] and to strengthen its interfacial bonding with the binder is a core challenge for improving the performance of ADN-based composite materials.

Graphene oxide (GO), as a representative of nanocarbon materials, is widely used for the reinforcement and functionalization of composite materials due to its unique two-dimensional structure, abundant surface functional groups, and excellent mechanical properties [15,16,17]. Introducing GO into the ADN-based composite system holds the potential to leverage its surface oxygen-containing groups to form strong non-covalent interactions, such as hydrogen bonds, with both ADN and the binder. This can microstructurally construct a physically cross-linked network, thereby achieving a synergistic effect of coating ADN, improving interfacial bonding, and simultaneously enhancing both strength and toughness [18,19].

Molecular dynamics (MD) simulation is a computational technique based on Newton’s laws of mechanics, primarily used to simulate and analyze the microscopic behavior and properties of substances. Chen et al. [20] used molecular dynamics to predict the interaction and compatibility between ADN and the most commonly used fluorine-containing binders in solid propellants. The results indicated that adding fluorine-containing polymers can effectively improve the mechanical properties of ADN, with polytetrafluoroethylene (PTFE) and fluorrubber 26 (F26) showing promise as binders in ADN-based solid propellants. Yu et al. [21] added four polymer binders, i.e., glycidyl azide polymer (GAP), polyglycidyl nitrate (PGN), polyethylene glycol (PEG) and polytetrahydrofuran (poly-THF), to dihydroxylammonium 5,5′-bistetrazole-1,1′-diolate (TKX-50) at mass fractions ranging from 1% to 12%, simulating the effects of binder mass fraction and structure on the mechanical properties of the system. They found that as the polymer mass fraction increased, the elastic modulus of the system decreased. PBXs containing polymers with side chains in their molecular structure (such as GAP and PGN) exhibited higher elastic moduli than those containing polymers without side chains (such as PEG and poly-THF). Fu et al. [22] reported the binding energy (*E_bind_*), cohesive energy density (CED), and isotropic mechanical properties of 1,1-diamino-2,2-dinitroethene (FOX-7) and its PBX at various temperatures. The results indicated that the binding energy exhibited a non-monotonic variation with increasing temperature. As the temperature rose, *L_max_* increased while CED decreased. The intermolecular interactions between FOX-7 and the polymer contributed to shortening the C-NO_2_ bond length, thereby reducing the mechanical sensitivity of FOX-7. To better investigate the interactions among ADN, GO, and the binder, molecular dynamics simulation [23,24,25] was employed to reveal the nature of interfacial interactions and the dynamic evolution of structure/property relationships at the molecular/atomic scale [26,27].

In this study, GO was utilized to function as a “dual-sided adhesive” between the energetic component ADN and the outer binder layer, forming an ADN/GO–binder double-shell structure. This design aims to protect the inner energetic material while enhancing adhesion through the surface binder. Molecular dynamics simulations were employed to investigate the compatibility and interaction mechanisms between different binders and the ADN/GO interface, as well as the molecular mobility and thermodynamic properties within the composite system. The findings provide a theoretical foundation and formulation guidance for designing high-performance, highly stable ADN-based composite energetic materials.

## 2. Results and Discussion

### 2.1. Prediction of Blend Compatibility

In the design of coating layers for composite energetic microspheres, the compatibility between the two components in the graphene oxide–binder (GO–binder) outer layer and the stability of the coating shell directly influence the overall material performance and the feasibility of the preparation process. To predict and optimize the formulation of this coating system, molecular dynamics simulations were first employed to evaluate the compatibility between the shell-layer GO and the binder. Specifically, the Blends module in MS was used to calculate the interaction parameter (*χ*) between them, thereby predicting their mixing compatibility and phase behavior. Based on the Flory–Huggins theory [28], this module simulates the structure and properties of polymer blends by obtaining the interaction parameter and volume fraction of the polymers, thus providing a theoretical basis for assessing the stability of the composite coating shell. In Flory–Huggins theory, the interaction parameter *χ* is defined as:

(1) $$\chi \;=\;\frac{E_{\mathrm{mix}}}{k_{b}T}$$

In the expression, *E_mix_* is the mixing energy, *k_b_* is the Boltzmann constant, and *T* is the temperature. Generally, a small or negative *χ* value indicates favorable affinity between the two components at a given temperature, whereas a large positive *χ* value suggests that the components are more likely to be attracted to their own kind rather than to mix with each other. When *χ* exceeds a certain critical value, the change in free energy during mixing overcomes the contribution of configurational entropy, leading to phase separation in the system.

**Figure 1:**
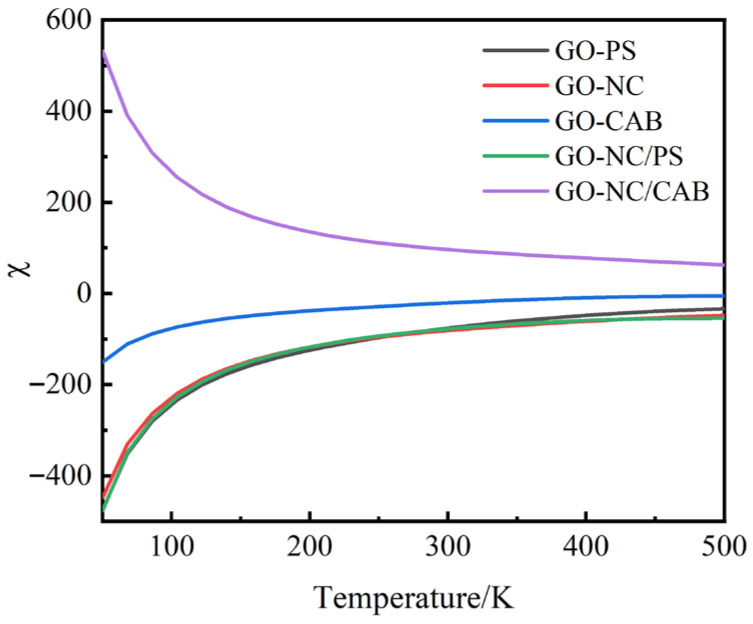
GO–binder (PS, NC, CAB, NC/PS, and NC/CAB) interaction parameter *χ*/temperature curve.

The *χ*/temperature curve depicting the interaction energy parameter between GO and the binder is shown in Figure 1. Analysis of the GO–binder interaction parameter *χ*/temperature curves provides clearer insight: as temperature increases, the *χ* values for all systems trend towards zero. This indicates that with rising temperature, the interaction between the two components strengthens, and mixing compatibility improves. Specifically, the *χ* values for the GO–NC/CAB system remain positive across the studied temperature range, suggesting that this system is thermodynamically incompatible and exhibits a tendency for macroscopic phase separation. For the GO–CAB, GO–PS, GO–NC, and GO–NC/PS systems, the *χ* values are consistently negative, indicating thermodynamic stability and good temperature adaptability across various ambient conditions relevant to practical applications. This suggests favorable compatibility between the components at the given temperatures.

**Figure 2:**
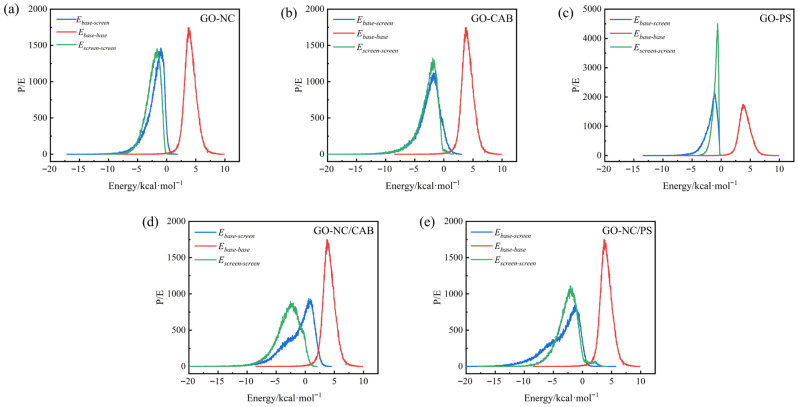
GO–binder binding energy distribution curve of (**a**) GO–NC, (**b**) GO–CAB, (**c**) GO–PS, (**d**) GO–NC/CAB, (**e**) GO–NC/PS.

The compatibility of blends can also be assessed using the binding energy distribution curves of the two components. If the shapes of the distribution curves are similar, it generally indicates that the two components mix readily and exhibit good compatibility. Conversely, significant differences in the distribution curves suggest poor compatibility. In this simulation, GO is defined as the base, and the binder is defined as the screen. The binding energy distribution curve between GO and the binder is presented in Figure 2.

As shown in Figure 2, the self-interaction energy of GO (*E_bb_*) in all systems is around +3.9 kcal·mol^−1^, indicating a stable repulsive interaction between GO sheets. This suggests that GO sheets are less likely to agglomerate in the composite material, favoring their dispersion in monolayer or few-layer forms. For the NC, CAB, PS, and NC/PS systems, the GO–binder interfacial energy (*E_bs_*) is negative, confirming the stability of the interfacial bonding. However, in the mixed binder system NC/CAB, the interfacial energy (*E_bs_*) shifts to a positive value, indicating thermodynamic repulsion between GO and the mixed binder. The self-cohesion energy (*E_ss_*) of the mixed binder is significantly enhanced compared to that of single binders, suggesting that the self-cohesive forces between binder molecules increase after mixing. This enhanced self-cohesion contributes to the formation of a binder network with certain toughness and strength.

The GO–NC/PS hybrid system exhibits a 1 + 1 > 2 synergistic effect. The rigid benzene rings of PS restrict the self-aggregation of NC, allowing more polar groups of NC to be exposed on the GO surface. Meanwhile, the polarity of NC enhances the polar environment at the PS-GO interface, thereby strengthening π-π stacking. PS anchors onto GO via π-π stacking, while NC anchors onto GO through hydrogen bonding. The simultaneous interaction of these two mechanisms on the GO surface increases the total strength of interfacial bonding.

### 2.2. Glass Transition Temperature (T_g_)

The flowability, stability, and other properties of polymers at specific temperatures are closely related to the glass transition temperature (*T_g_*), which serves as a key indicator of their thermal performance [29,30]. The *T_g_* is the characteristic temperature at which a polymer transitions from the glassy state to the rubbery state. A higher *T_g_* implies that the material can maintain greater stiffness and strength over a higher temperature range. Therefore, accurately predicting the *T_g_* of the GO–binder coating layer is crucial for ensuring the structural integrity and thermal stability of ADN/GO–binder composite energetic materials after forming.

The constructed GO model and five types of binders (NC, CAB, PS, NC/CAB, NC/PS) were assembled into a layered structure. Structural optimization was performed using the Dreiding force field and the Smart algorithm. The maximum number of iteration steps was set to 50,000, and the external pressure was set to 10^−4^ GPa. The initial atomic velocities were assigned randomly according to a Gaussian distribution, and the force field type for each atom was assigned manually. Molecular dynamics calculations were conducted to fully relax the optimized structure. The simulation was carried out in the NVT ensemble at a temperature of 298 K, with temperature control achieved via the Velocity Scale method [31]. The simulation time step was set to 1 fs, with a total simulation time of 500 ps. Subsequently, the relaxed GO–binder model was further simulated for 500 ps in the NPT ensemble, maintaining the temperature at 298 K (controlled by Velocity Scale) and applying a pressure of 10^−4^ GPa (controlled by the Parrinello method) [32]. The Velocity Scale thermostat essentially introduces a random force based on the Berendsen thermostat, thereby addressing the issue that particle velocities do not satisfy the Maxwell distribution while retaining its advantages [33]. The Parrinello–Rahman barostat employs an extended Lagrangian method similar to the Nosé–Hoover thermostat, enabling the correct generation of the NPT ensemble. Electrostatic interactions were calculated using the Ewald summation method, while van der Waals interactions were treated with the atom-based summation method. Based on this, molecular dynamics calculations were further performed under the NVT ensemble to finally equilibrate the system, obtaining a fully equilibrated structural model with the correct density for subsequent *T_g_* simulation calculations, with a total simulation duration of 1 ns.

**Figure 3:**
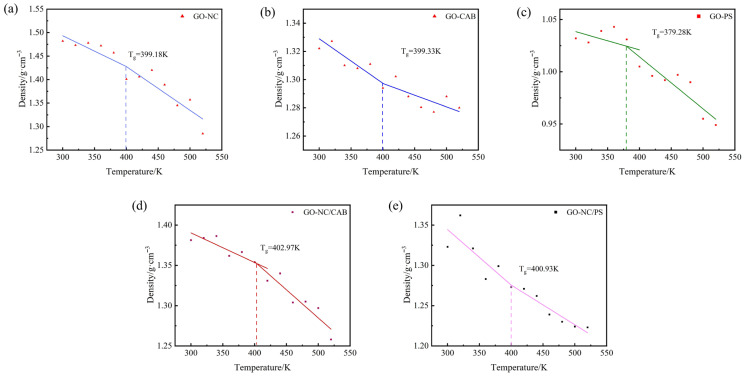
The *ρ*-*T* fitting curve of (**a**) GO–NC, (**b**) GO–CAB, (**c**) GO–PS, (**d**) GO–NC/CAB, (**e**) GO–NC/PS.

The *T_g_* was determined via molecular dynamics simulation, with a complete “cooling down” process being the central step in the procedure [29,30,34]. For each equilibrated system, the temperature is first gradually increased from 298 K to 520 K through annealing to smoothly raise the system to the desired temperature. Subsequently, the system is cooled stepwise from 520 K, with each step decreasing the temperature by 20 K, undergoing a total of 12 cooling cycles. Each cycle consists of 350 ps of molecular dynamics simulation and 300 ps of NPT simulation for equilibration, followed by 50 ps of NVT simulation for data sampling to obtain the average density (*ρ*) of the system. The final equilibrated configuration from each step is used as the initial configuration for the next cycle. By recording the density and temperature (*T*) of the system during the cooling process, a *ρ*-*T* scatter plot was ultimately generated, which illustrates the variation in density with decreasing temperature. The glass transition temperature *T_g_* of the material is determined by performing linear fitting on the scatter plot and identifying the intersection point of the fitted lines. The fitted *ρ*-*T* curves for each system are shown in Figure 3.

The *T_g_* was determined from the intersection point of the *ρ*-*T* fitting curves. And the *T_g_* values for the shell layers formed by GO–NC, GO–CAB, GO–PS, GO–NC/CAB, and GO–NC/PS are 399.18 K, 399.33 K, 379.28 K, 402.97 K, and 400.93 K, respectively. The results indicate that, compared to the single-binder layers (GO–NC, GO–CAB, GO–PS), the composite binders (GO–NC/CAB, GO–NC/PS) exhibit higher glass transition temperatures, indicating that their shell layers require greater activation energy to initiate slip or flow. From a macroscopic perspective, this demonstrates that composite binders, when used as shell layers, offer better mechanical performance and encapsulation stability at low temperatures. Additionally, a higher glass transition temperature reflects stronger intermolecular interactions within the polymer and a more ordered molecular arrangement, which contributes to enhancing the mechanical properties of the material.

### 2.3. Binding Energy

To characterize interfacial binding strength, interaction energy and binding energy are commonly employed as key indicators [35,36]. In this work, by calculating the interaction energy per unit area (*E′_inter_*) and the binding energy (*E_bind_*) for the ADN/GO–binder and ADN-binder systems, we aim to quantify the interfacial adhesion between different binders and GO surface-modified ADN, and to elucidate whether GO modification exerts an enhancing effect on the interface between ADN and the binder. The calculation formulas are as follows:

(2) $$E_{\mathrm{inter}}\;={\;E}_{\mathrm{total}\;}-{\;E}_{1}\;-{\;E}_{2}$$

(3) $$E_{\mathrm{inter}}^{\prime }=\frac{E_{\mathrm{inter}}}{A_{12}}$$

(4) $$E_{\mathrm{bind}}=\;-E_{\mathrm{inter}}$$

In the formulas, *E_total_* is the total energy of the system, *E*_1_ denotes the energy of the ADN/GO component within the system, *E*_2_ signifies the energy of the binder component, and *A*_12_ indicates the cross-sectional area of the interfacial region where interaction occurs. The binding energy is the negative value of the interaction energy.

For the five constructed ADN/GO–binder systems, force fields were manually assigned to each atom. Structural optimization was performed under the Dreiding force field. Subsequently, a 500 ps molecular dynamics simulation was performed in the NVT ensemble to allow the structures to fully relax. The relaxed structures then underwent a 500 ps molecular dynamics simulation in the NPT ensemble, followed by an additional 500 ps calculation in the NVT ensemble based on this. During the simulation, the temperature was set to 298 K and controlled using the Velocity Scale method, while the pressure was maintained at 10^−4^ GPa with the Parrinello barostat. The total simulation duration was 1.5 ns with a time step of 1 fs. Ultimately, the equilibrated structures of each system were obtained for subsequent computational analysis. The calculation results are presented in Table 1.

**Figure 4:**
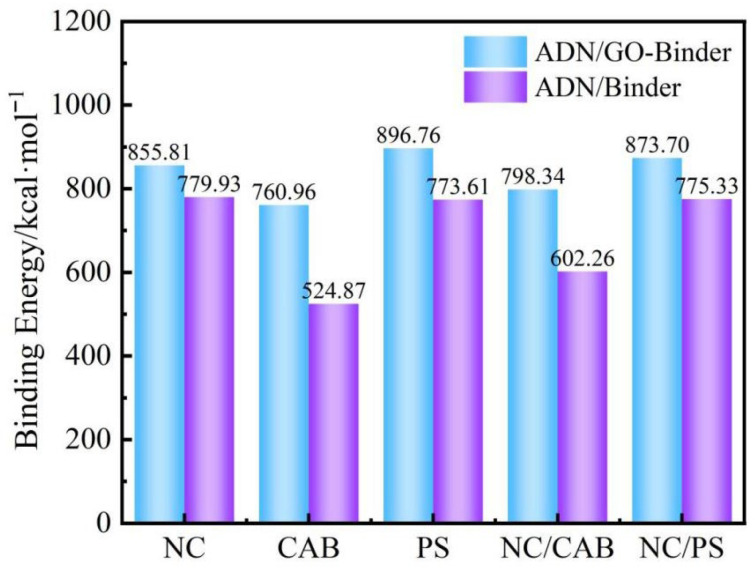
Binding energy between ADN/GO–binder (NC, CAB, PS, NC/CAB, NC/PS) and ADN-binder (NC, CAB, PS, NC/CAB, NC/PS).

Binding energy can be understood as the energy released when different molecules approach each other, resulting in a decrease in the total energy of the system. This energy represents an attractive interaction. As shown in Figure 4, compared to the direct interfacial interaction between ADN itself and the outer binder, the interaction energy and binding energy between GO surface-modified ADN/GO and the binder are both enhanced. This demonstrates that GO, as an intermediate layer, acts as a “double-sided adhesive”, improving its adhesion to the outer binder. The addition of GO shows the most pronounced enhancement effect at the CAB-ADN interface. However, the interaction energy between NC/PS and ADN consistently remains at a relatively high level.

### 2.4. Radial Distribution Function (RDF)

The RDF can be used to characterize the microstructure of materials, reflecting the ratio of the probability density of finding another molecule around a given molecule to that of a random distribution [37]. In this study, intermolecular RDF analysis was performed for the ADN/GO–binder systems, with C and O atoms in GO selected as reference atoms and H atoms in ADN and the binder as target atoms. The function *g*(*r*) represents the ratio of the density of target H atoms within a distance *r* from the reference atoms (C, O) to the average density of the target H atoms in the entire system. The RDF can, to some extent, reflect the interaction characteristics between different atoms in the condensed phase.

**Figure 5:**
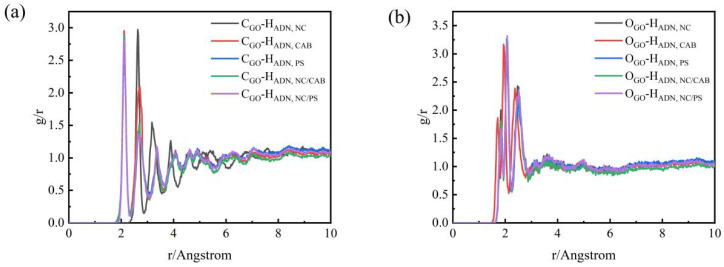
RDF of (**a**) C_GO–_H_ADN,binder_ and (**b**) O_GO–_H_ADN,binder_; binders are NC, CAB, PS, NC/CAB, NC/PS.

Analysis of Figure 5 shows that the *g*(*r*) for C_GO–_H_ADN,binder_ and O_GO–_H_ADN,binder_ exhibits peaks within the range of 1.7 Å to 2.5 Å, which corresponds to the hydrogen bonding range. This indicates that hydrogen bonding interactions exist between the C and O atoms of the intermediate GO layer and the H atoms from both ADN and the outer binder. The height of the peaks in each curve, to some extent, reflects the strength of the interactions. From Figure 5a, it can be seen that the hydrogen bonding between the C atoms of GO and the H atoms of ADN and NC is particularly strong in the ADN/GO–NC-PS system. This may be attributed to π–π stacking formed between the benzene rings of PS and GO, which brings ADN closer to the GO surface and draws the C–H groups near the nitro groups of NC closer to the GO surface, thereby enhancing π–H interactions. From Figure 5b, it is evident that the hydrogen bonding between the O atoms of GO and the H atoms of ADN and NC is strongest in the ADN/GO–NC/PS system. This is because NC provides numerous nitro group oxygen atoms, which can act as hydrogen bond acceptors, while ADN itself contains multiple N-H/O-H groups that can simultaneously form multiple hydrogen bonds with both GO–O and NC-O.

In summary, the GO–NC/PS system achieves the strongest intermolecular interactions among ADN, GO, and the binder through multiple mechanisms such as π-π stacking and multiple hydrogen bonds, thereby enhancing the interfacial bonding strength, thermal stability, mechanical properties, and energy transfer efficiency of the composite material.

### 2.5. Mean Square Displacement (MSD)

During the dynamic equilibrium process, molecules, polymer chains, and other components within the system undergo vibrations and trajectory changes over the simulation time. The mean square displacement (MSD) is a function of time. It measures the average squared displacement of particles over time *t*. The slope of the MSD curve directly reflects the diffusion capability of the particles; a larger slope and a steeper curve indicate stronger particle mobility within the system. The calculation formulas are as follows:

(5) $$\mathrm{MSD}\left(t\right)\;=\left\langle {\left|r\left(t\right)\;-\;\gamma \left(0\right)\right|}^{2}\right\rangle$$

(6) $$6D=\underset{t\to \infty }{\lim}\frac{\mathrm{MSD}\left(t\right)}{t}$$

In the equations, *r*(*t*) represents the displacement of a particle over time *t*, and the diffusion constant *D* (the diffusion coefficient) is obtained by fitting the slope of the MSD curve.

**Figure 6:**
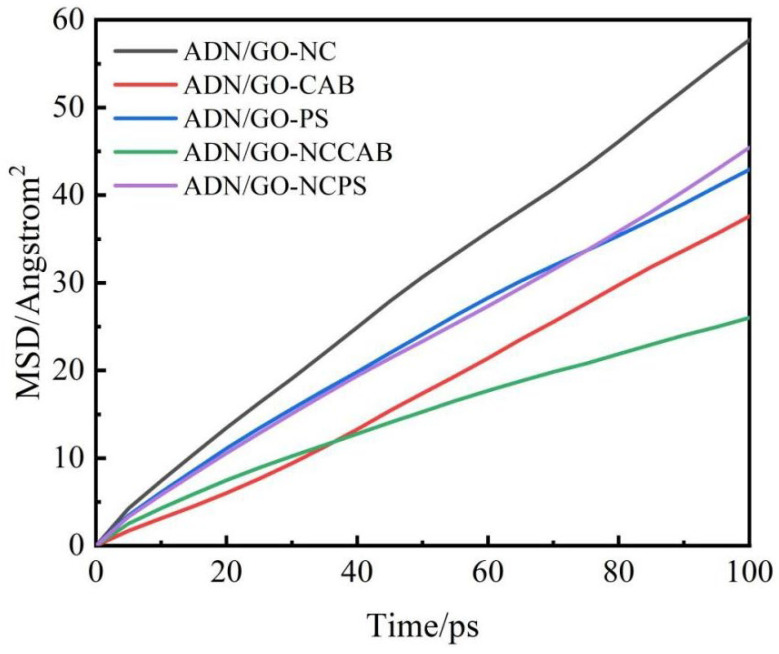
MSD curves of shell layer binders in each system (ADN/GO–NC, ADN/GO–CAB, ADN/GO–PS, ADN/GO–NCCAB, ADN/GO–NCPS).

Using the Forcite module, select Mean Square Displacement from Forcite Analysis for calculation. The MSD analysis was performed on the binder in the outer shell of the systems, with the results shown in Figure 6. The relatively flat MSD curves of the ADN/GO–NC/CAB and ADN/GO–CAB systems indicate that the binder chains within these systems exhibit weaker diffusion capability and restricted motion. This property may help prevent the material from deforming easily under external forces. In contrast, the steeper MSD curve of the ADN/GO–NC system reflects more frequent vibrational displacement of particles during equilibrium, which further demonstrates that intermolecular interactions in this system are weaker compared to others, potentially compromising long-term storage stability and safety performance. The ADN/GO–NC/PS and ADN/GO–PS systems occupy an intermediate position, indicating that their performance strikes a balance. They possess a certain level of rigidity to resist deformation under external forces while also reducing the likelihood of component separation, crystal growth, or internal stress generation during storage and use, thereby enhancing the safety and reliability of the material.

### 2.6. Cohesive Energy Density (CED)

Cohesive energy is defined as the energy required to overcome intermolecular forces and move aggregated matter beyond the range of molecular attraction [38,39]. The cohesive energy per unit volume is termed the CED. It is inherently a type of non-bonding interaction used to characterize the weak intermolecular interactions (van der Waals forces and electrostatic forces) within a system. It also serves as one of the metrics for evaluating system stability in interfacial studies.

**Figure 7:**
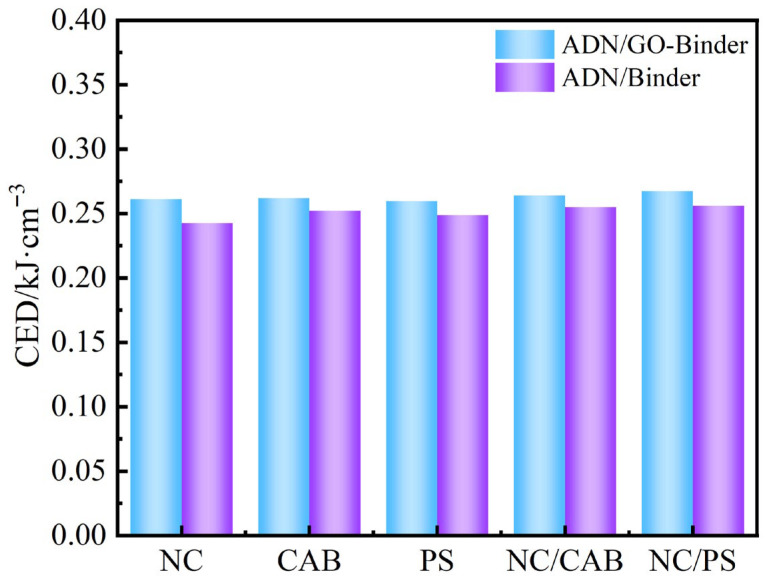
CED of ADN/GO–binder (NC, CAB, PS, NC/CAB, NC/PS) and ADN-binder (NC, CAB, PS, NC/CAB, NC/PS).

Using the Forcite module, select Cohesive Energy Density for calculation. CED calculations and analyses were performed for both the ADN/GO–binder and ADN/binder systems, with the results shown in Figure 7. After surface modification of ADN using GO as an intermediate layer, the CED of all systems increased. This indicates that the incorporation of GO enhanced the interaction between ADN and the binder to some extent across different binder systems.

For the single-binder systems, the CED values of GO–NC and GO–CAB are similar and both are higher than that of GO–PS. This is because NC and CAB contain abundant polar groups (e.g., nitro, hydroxyl, and ester groups), which can form strong hydrogen bonds and dipole interactions with the oxygen-containing functional groups on the GO surface, thereby enhancing the overall cohesive energy. In contrast, PS possesses a hydrophobic aromatic ring structure and exhibits weaker affinity with the polar interface of GO, with intermolecular interactions dominated by weak van der Waals forces, resulting in the lowest CED.

For the composite binder systems, the CED values of both GO–NC/CAB and GO–NC/PS are higher than those of any single-binder system, demonstrating a synergistic enhancement effect. NC and CAB are both cellulose derivatives, capable of forming hydrogen bonds and achieving tight interchain packing, thereby enhancing the CED. For NC/PS, polar interactions occur between the strongly polar nitro groups of NC and the benzene rings of PS. Meanwhile, the rigid chain segments of PS can induce local ordered arrangement of NC, further increasing intermolecular packing density and leading to the highest CED.

### 2.7. Mechanical Property

Excellent mechanical properties are crucial for ensuring the structural integrity and safety of explosives during loading, transportation, and service. Based on molecular dynamics methods, the regulatory effects of different binders on the mechanical properties of ADN/GO were systematically evaluated.

The Forcite module was employed to compute the mechanical properties. According to Voigt theory [33], the formulas for calculating the bulk modulus (*K_V_*) and shear modulus (*G_V_*) of the composite system’s layered structure, and the formulas for Young’s modulus (*E*) and Poisson’s ratio (*v*) are as follows [40]:

(7) $$9K_{V}=\;(C_{11}\;+\;C_{22}\;+\;C_{33})\;+\;2(C_{12}\;+\;C_{23}\;+\;C_{13})\;$$

(8) $$15G_{V}=(C_{11}+C_{22}+C_{33})\;-\;(C_{12}+C_{23}+C_{13})+3(C_{44}+C_{55}+C_{66})$$

(9) $$\nu =\frac{1}{2}(1\;-\;\frac{3G}{3K+G})$$

(10) $$\frac{1}{E}=\frac{1}{3G}+\frac{1}{9K}$$

Based on this, the elastic constants and mechanical properties of each system were calculated, as shown in Table 2. Young’s modulus is related to a material’s ability to resist elastic deformation; a smaller value indicates better elasticity. Shear modulus reflects plasticity, with a smaller value suggesting better plasticity. Bulk modulus is used to correlate with the fracture strength of a material, where a larger value indicates higher fracture strength. The ratio *K*/*G* represents the ratio of bulk modulus to shear modulus and is used to indicate a material’s ability to withstand strong impacts without failure; a larger value indicates greater toughness. The Cauchy pressure (*C*_12_–*C*_44_) can be used to measure the ductility of a material. A negative value indicates brittleness, while a positive value suggests better ductility.

Compared to the ADN/GO system, the addition of any binder (NC, CAB, PS, NC/CAB, NC/PS) significantly improved the elastic constants and macroscopic moduli of the composite material. This indicates that the binders effectively enhanced interfacial bonding within the system, promoted stress transfer, and thereby increased overall stiffness and strength. The Poisson’s ratio *ν* showed minimal variation, suggesting that the binder had a limited impact on the material’s compressibility. CAB and PS were the most prominent in increasing Young’s modulus (*E*), reaching 126.120 GPa and 126.588 GPa, respectively. If the process requires the shell layer to maintain high resistance to deformation during pressing, priority should be given to CAB or PS. However, GO–CAB exhibits the most negative Cauchy pressure, indicating the strongest tendency toward brittleness. Its high modulus comes at the expense of toughness, making it prone to crack initiation during processing and under impact. NC contributed to higher bulk modulus (*K*) and *K*/*G* ratios, indicating its role in enhancing compressive resistance and moderate toughness. For mixed binders, NC/CAB and NC/PS exhibited intermediate performance compared to single binders but demonstrated better overall comprehensive properties. In particular, while the Young’s modulus of the NC/PS system was slightly lower than that of CAB and PS, its other performance indicators were more balanced. The shear elastic constants *C*_44_, *C*_55_, and *C*_66_ of the GO–NC/PS system were 47.767, 48.178, and 47.343 GPa, respectively, showing similar and relatively high values. This indicates that the material’s resistance to deformation was relatively consistent across different shear directions, in contrast to PS, which significantly increased *C*_66_ but resulted in slightly lower values for other shear constants. The *K*/*G* ratio of GO–NC/PS was 1.397, comparable to that of CAB, suggesting that it maintained appropriate toughness while improving stiffness, which is beneficial for the safety and reliability of energetic materials. This may be attributed to the favorable adhesion and mechanical strength of NC, combined with the high stiffness and processability provided by PS. When these two components interact synergistically, they likely form a more uniform and stable transitional phase at the ADN/GO interface, enhancing interfacial bonding strength and stress transfer efficiency, thereby improving the overall mechanical performance. Furthermore, its *C*_12_–*C*_44_ value of −18.101 falls within a moderate range, indicating good structural stability.

In summary, binders effectively modulate the mechanical properties of ADN/GO composite materials by enhancing interfacial interactions. Among them, the GO–NC/PS system achieves the best balance among stiffness, toughness, isotropy, and shear performance due to the synergistic effect of NC and PS, thereby demonstrating superior overall performance. This synergy results in a 1 + 1 > 2 enhancement effect, making it an ideal candidate for the structural design of energetic materials.

## 3. Modeling and Simulation

### 3.1. Model Construction

**Figure 8:**
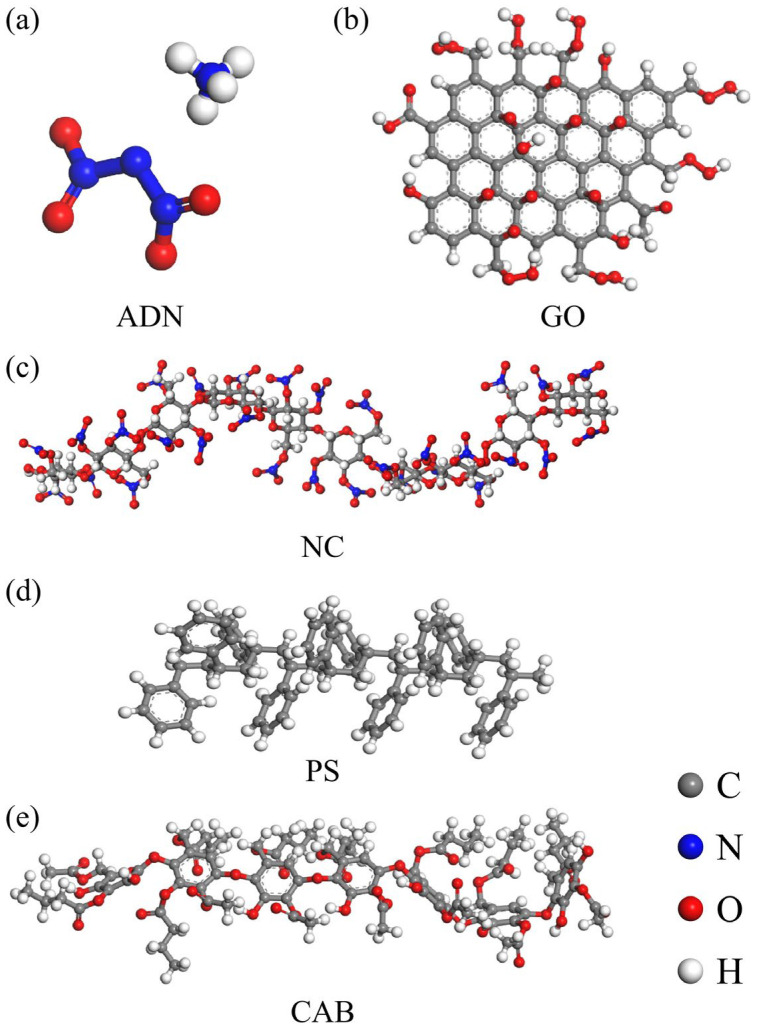
Molecular model of (**a**) ADN, (**b**) GO, (**c**) NC, (**d**) PS, (**e**) CAB.

The initial crystal structure of ADN was sourced from the Cambridge Crystallographic Data Centre (CCDC) [41]. The GO model was constructed based on the carbon hexagonal ring framework of graphene. A single-layer graphene sheet was first generated using the Build module in MS. Oxygen-containing functional groups were then added to this base structure, and the bonding configurations were adjusted, as illustrated in Figure 8b. For the outer coating material, binders including NC, CAB, PS, and NC/PS and NC/CAB were selected for investigation, as illustrated in Figure 8c–e. The polymer chains for each binder were constructed using the Build polymer tool.

**Figure 9:**
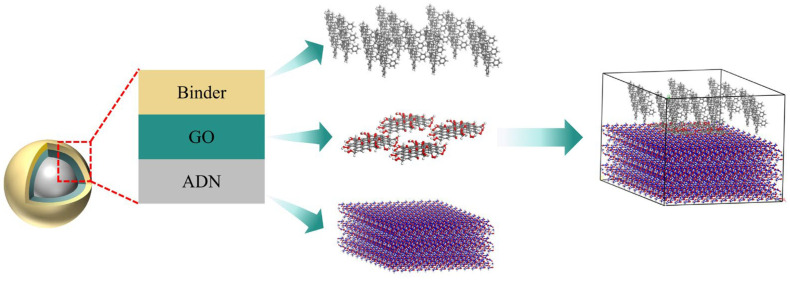
Schematic diagram of ADN/GO–binder layered model construction.

To more accurately simulate interfacial interactions, the model was constructed with GO accounting for 1% and the binder for 5% by proportion. Using the Amorphous Cell module, a corresponding ADN/GO–binder layered model was built by combining the ADN supercell with the previously constructed GO and binder models. A vacuum layer with a thickness of 30 Å was included, with the layers arranged sequentially as ADN, GO, and binder. The energetic component ADN and the polymer chains represent the microscopic scale, while the layered model exists at the mesoscale. This mesoscale study aims to reveal the correlations with interfacial interactions at the macroscopic scale, where binder-coated GO surface-modified ADN particles are involved. A schematic diagram of the layered model construction is shown in Figure 9 below.

### 3.2. Molecular Dynamics Simulation

(1) Model Construction

The initial configuration of the ADN/GO–binder blend system was generated using the AC module, and geometric optimization of the structure was subsequently performed with the Forcite module.

(2) Simulation Conditions

The Dreiding force field was adopted throughout the simulation, and atomic charges were assigned via the Gasteiger method [42,43,44]. Molecular dynamics simulations were carried out in the NVT ensemble for 100 ps at a temperature of 298 K, employing a time step of 1 fs and an external pressure of 10^−4^ GPa. Temperature control was achieved using the Nosé thermostat, while pressure was regulated by the Berendsen barostat.

(3) Simulation Outcomes

Electrostatic interactions were evaluated using the Ewald summation technique, and van der Waals interactions were computed with the atom-based summation method. Through the aforementioned optimization and simulation procedures, a thermodynamically equilibrated model of the ADN/GO–binder blend system was successfully obtained. Subsequently, the blend compatibility, *T_g_*, binding energy, RDF, MSD, CED, and mechanical property were systematically investigated and analyzed.

To verify the reproducibility of the molecular simulation methodology, all calculations were independently repeated three times, and the results demonstrated excellent consistency across all replicate runs.

## 4. Conclusions

This study systematically investigated the microstructure, thermodynamic properties, and mechanical performance of ADN-based composite materials using molecular dynamics simulations, with GO as a critical intermediate layer and different polymer binders as the outer shell. This research confirms that introducing GO as a molecular double-sided adhesive and barrier layer, combined with the rational selection of binders, is an effective strategy for achieving high-performance ADN-based composite systems. The simulation results indicate that the chemical nature of the binder is the key determinant of the composite system’s performance.

(1) Using MS, a systematic study was conducted on the ADN/GO–binder composite systems with NC, CAB, PS, and their blends NC/CAB and NC/PS as binders. Among these, the NC/PS binder demonstrated the best performance.
(2) The GO–NC/PS system exhibits a negative Flory–Huggins interaction parameter, indicating thermodynamic stability at the interface. Its glass transition temperature is as high as 400.93 K, suggesting better stability and reliability across a wide temperature range.
(3) RDF analysis reveals that van der Waals forces and hydrogen bonding exist between different binder systems and ADN. In particular, the NC/PS composite system achieves the strongest intermolecular interactions among ADN, GO, and the binder through multiple mechanisms, including π–π stacking and multiple hydrogen bonds.
(4) The GO–NC/PS system shows relatively low anisotropy in its elastic constants, indicating more consistent mechanical responses in different directions. Its moderate Cauchy pressure and K/G ratio suggest that it maintains relatively good structural stability while achieving high stiffness, which is beneficial for the safe application of energetic materials.
(5) The combination of NC and PS is not a simple additive effect but produces a synergistic enhancement where the whole is greater than the sum of its parts. NC provides strong polarity and adhesion, while PS contributes high stiffness and π-π stacking capability. Together, they form a stronger cohesive energy network at the GO interface, enabling efficient energy transfer and stress distribution among ADN, GO, and the binder through multiple interactions.

## Figures and Tables

**Table 1:** Interaction energy and interaction energy per unit area between ADN/GO–binder (NC, CAB, PS, NC/CAB, NC/PS) and ADN-binder (NC, CAB, PS, NC/CAB, NC/PS).

Systems	Interaction Energy (kcal/mol)	Interaction Energy per Unit Area (kcal/mol·Å^2^)	Systems	Interaction Energy (kcal/mol)	Interaction Energy per Unit Area (kcal/mol·Å^2^)
ADN/GO–NC	−855.81	−0.11	ADN/NC	−779.93	−0.10
ADN/GO–CAB	−760.96	−0.12	ADN/CAB	−524.87	−0.09
ADN/GO–PS	−896.76	−0.15	ADN/PS	−773.61	−0.13
ADN/GO–NC-CAB	−798.34	−0.12	ADN/NC-CAB	−602.26	−0.09
ADN/GO–NC-PS	−873.70	−0.23	ADN/NC-PS	−775.33	−0.21

**Table 2:** The elastic constant matrices (*Cij*) and mechanical properties of the ADN/GO, ADN/GO–NC, ADN/GO–CAB, ADN/GO–PS, ADN/GO–NC-CAB, ADN/GO–NC-PS systems.

Constants	ADN/ GO	ADN/ GO–NC	ADN/ GO–CAB	ADN/ GO–PS	ADN/ GO–NC-CAB	ADN/ GO–NC-PS
*C*_11_	119.517	176.317	187.448	180.803	182.087	182.211
*C*_22_	118.301	127.743	125.029	130.579	125.916	127.712
*C*_33_	120.433	129.707	137.877	133.642	134.369	131.264
*C*_44_	44.117	47.019	48.225	48.805	47.909	47.767
*C*_55_	44.325	46.784	49.387	49.414	48.216	48.178
*C*_66_	43.612	46.494	46.97	50.191	46.778	47.343
*C*_12_	30.729	29.075	29.342	31.652	29.589	29.666
*C*_13_	31.169	38.522	40.821	40.593	39.713	39.525
*C*_23_	31.152	32.831	33.991	34.368	32.831	33.441
*K* (GPa)	60.483	70.514	73.185	73.139	71.849	71.828
*G* (GPa)	44.091	50.282	51.996	52.243	51.263	51.228
ν	0.207	0.212	0.213	0.212	0.212	0.212
*E* (GPa)	106.415	121.877	126.120	126.588	124.241	124.165
*K*/*G*	1.372	1.402	1.397	1.393	1.401	1.397
*C*_12_–*C*_44_ (GPa)	−13.388	−17.944	−18.883	−17.153	−18.32	−18.101

## Data Availability

The original contributions presented in this study are included in the article. Further inquiries can be directed to the corresponding author.
